# Efficacy of Different Beta Blockers in Reducing Mortality in Heart-Failure Patients

**DOI:** 10.7759/cureus.74171

**Published:** 2024-11-21

**Authors:** Salman Habib Roghani, Dr Sanaullah Khan, Aatika Shafiq, Amna Akbar, Waqar Mustafa, Syed Qamber Ali Shah, Marriam Khan, Hasnain Ali

**Affiliations:** 1 Cardiology, Pakistan Institute of Medical Sciences, Islamabad, PAK; 2 Internal Medicine, Fatima Jinnah Medical University, Lahore, PAK; 3 Emergency and Accident, District Headquarter Hospital, Jhelum Valley, Muzaffarabad, PAK; 4 Cardiology, Abbas Institute of Medical Sciences, Muzaffarabad, PAK; 5 General Medicine, Royal Bournemouth Hospital, University Hospitals Dorset NHS Foundation Trust, Bournemouth, GBR; 6 Health, Indus Hospital, Karachi, PAK; 7 Medicine, Army Medical College, Rawalpindi, PAK

**Keywords:** beta blockers, bisoprolol, carvedilol, heart failure, heart failure hospitalization, left ventricle ejection fraction, mortality

## Abstract

This study evaluated the comparative efficacy of different beta blockers bisoprolol, carvedilol, and metoprolol in reducing mortality and hospitalizations among 120 heart-failure (HF) patients. The sample had an equal gender distribution (50% male, 50% female) with a mean age of 69.28 years. Baseline characteristics, such as systolic blood pressure (mean: 134.36 mmHg) and left ventricular ejection fraction (LVEF) (mean: 40.24%), were comparable across the treatment groups. Patients were treated with either bisoprolol (30%), carvedilol (30%), or metoprolol (40%) for an average of 27.54 weeks. The study utilized Poisson and negative binomial regression models to assess hospitalization rates, and chi-square tests to compare mortality outcomes. Results revealed that mortality was 44.2% across the entire cohort, with no significant differences between the three beta-blocker groups (p = 0.301). Similarly, no significant differences were observed in hospitalizations (p = 0.276) or ICU admissions (p = 0.797). However, patients on bisoprolol and carvedilol exhibited a slight improvement in New York Heart Association (NYHA) class and LVEF, though this was not statistically significant (p = 0.145 and p = 0.477, respectively). Side effects, including bradycardia, fatigue, and hypotension, were noted in 32.5%, 21.7%, and 23.3% of patients, respectively. These findings suggest that all three beta blockers are similarly effective in reducing mortality, though bisoprolol and carvedilol may offer better control of HF symptoms.

## Introduction

Heart failure (HF) is a significant global health issue, affecting millions of individuals worldwide. It is characterized by the heart's inability to pump sufficient blood to meet the body's needs, leading to a range of symptoms such as shortness of breath, fatigue, and fluid retention [1,2]. Despite advances in treatment, HF continues to be a leading cause of morbidity and mortality, with high hospitalization rates and poor survival, particularly in advanced stages of the disease [3,4]. Among the primary pathophysiological mechanisms contributing to HF is the chronic activation of the sympathetic nervous system, which, while initially compensatory, leads to harmful long-term effects on cardiac function. In this context, bet blockers have become a cornerstone of therapy, particularly in HF with reduced ejection fraction (HFrEF) [5,6]. Beta blockers inhibit the effects of catecholamines on beta-adrenergic receptors, helping to reduce heart rate, myocardial oxygen demand, and the adverse consequences of chronic sympathetic activation. Clinical trials have demonstrated their ability to improve symptoms, reduce hospitalizations, and, most importantly, lower mortality rates in HF patients [7]. However, questions remain regarding the comparative efficacy of different beta blockers, sparking ongoing debate among clinicians about which specific drug provides the most significant survival benefit in this patient population [8,9].

The etiology of HF involves complex neurohormonal interactions, with particular focus on the sympathetic nervous system and the renin-angiotensin-aldosterone system (RAAS) [10,11]. In patients with HF, chronic overactivation of the sympathetic nervous system leads to elevated levels of circulating catecholamines, such as norepinephrine, which initially help maintain cardiac output by increasing heart rate and contractility. Over time, however, this persistent sympathetic drive results in increased myocardial oxygen demand, arrhythmias, and progressive remodeling of the heart, ultimately exacerbating the underlying HF [12,13]. Beta blockers work by antagonizing the effects of these catecholamines on beta-adrenergic receptors, reducing heart rate, lowering myocardial contractility, and preventing arrhythmias. Additionally, they have been shown to inhibit ventricular remodeling, a key pathological process that drives worsening of HF [14]. By mitigating these harmful effects, beta blockers provide a survival benefit in HF patients. Several beta blockers, including carvedilol, metoprolol succinate, bisoprolol, and nebivolol, have been extensively studied in clinical trials and are recommended by current guidelines for HF treatment. However, differences in their pharmacological properties have led to questions about whether certain beta blockers may offer greater mortality reduction than others. For example, carvedilol, with its combined beta and alpha-blocking properties, may offer additional vasodilatory effects compared to more selective agents like bisoprolol and metoprolol, which predominantly target beta-1 receptors in the heart.

A substantial body of literature supports the use of beta blockers in HF, with landmark trials such as CIBIS-II (1999), MERIT-HF (1999), and COPERNICUS (2001) establishing their role in reducing mortality and improving clinical outcomes in patients with HFrEF. The CIBIS-II trial, which compared bisoprolol to placebo, found a significant reduction in all-cause mortality among HF patients receiving bisoprolol. Similarly, the MERIT-HF trial demonstrated that metoprolol succinate reduced all-cause mortality and hospitalizations when compared to placebo. The COPERNICUS trial showed that carvedilol provided a substantial mortality and morbidity reduction in patients with severe HF. These trials have firmly established the role of beta blockers in HF therapy [15]. However, they have also raised important questions about the comparative efficacy of different beta blockers. Carvedilol, with its additional alpha-blocking effects, may provide enhanced vasodilatory benefits, while cardioselective agents such as metoprolol and bisoprolol are thought to offer more targeted action with potentially fewer side effects, such as bronchospasm. Some studies have found minimal differences between beta blockers in terms of clinical outcomes, suggesting that the choice of agent may depend more on individual patient characteristics, tolerability, and comorbid conditions [16].

The primary aim of this research is to compare the efficacy of different beta blockers in reducing mortality among HF patients. The study will focus on the most widely used beta blockers in HF management, including carvedilol, metoprolol succinate, bisoprolol, and nebivolol [17]. Specifically, this research aims to evaluate the relative mortality reduction associated with each beta blocker, assess the safety and tolerability profiles of these drugs, and determine whether patient characteristics such as age, comorbidities, or HF severity influence the efficacy of beta blocker therapy. Additionally, this study will investigate whether the selectivity of beta blockers (cardioselective vs. non-selective) impacts clinical outcomes, particularly mortality. By conducting a comparative analysis of these agents, this research seeks to provide clinical recommendations that will help healthcare providers select the optimal beta blocker therapy for HF patients, thereby improving patient outcomes and reducing the overall burden of HF on healthcare systems.

## Materials and methods

The study employs a retrospective cohort design to compare the efficacy of three beta blockers on mortality rates in HF patients. A total of 120 HF patients were selected from hospital records, ensuring equal representation of both male and female patients. Data collection included demographic variables (age, gender, ethnicity), clinical parameters (BMI, blood pressure, heart rate), comorbidities, and laboratory values (e.g., creatinine, cholesterol, sodium, and potassium levels). The treatment details, including the type of beta blocker, dosage, frequency of administration, treatment duration, and adverse effects, were also recorded. The primary outcome was mortality status, while secondary outcomes included ICU admissions and hospital visits. Statistical analysis was performed using SPSS, with descriptive statistics (mean, median, SD) calculated for continuous variables, and chi-square tests used to compare categorical variables such as gender and comorbidities. The Mann-Whitney U test was applied to compare continuous variables across groups, and a one-sample chi-square test was used to assess the distribution of categorical variables. The study adhered to ethical guidelines, and all patient data were anonymized. The analysis focused on comparing the efficacy of the beta blockers based on mortality outcomes, adjusting for potential confounders such as comorbidities, treatment duration, and dosage levels. The significance threshold was set at p<0.05, and all analyses were conducted with 95% confidence intervals.

## Results

Patients characteristics

**Figure 1:**
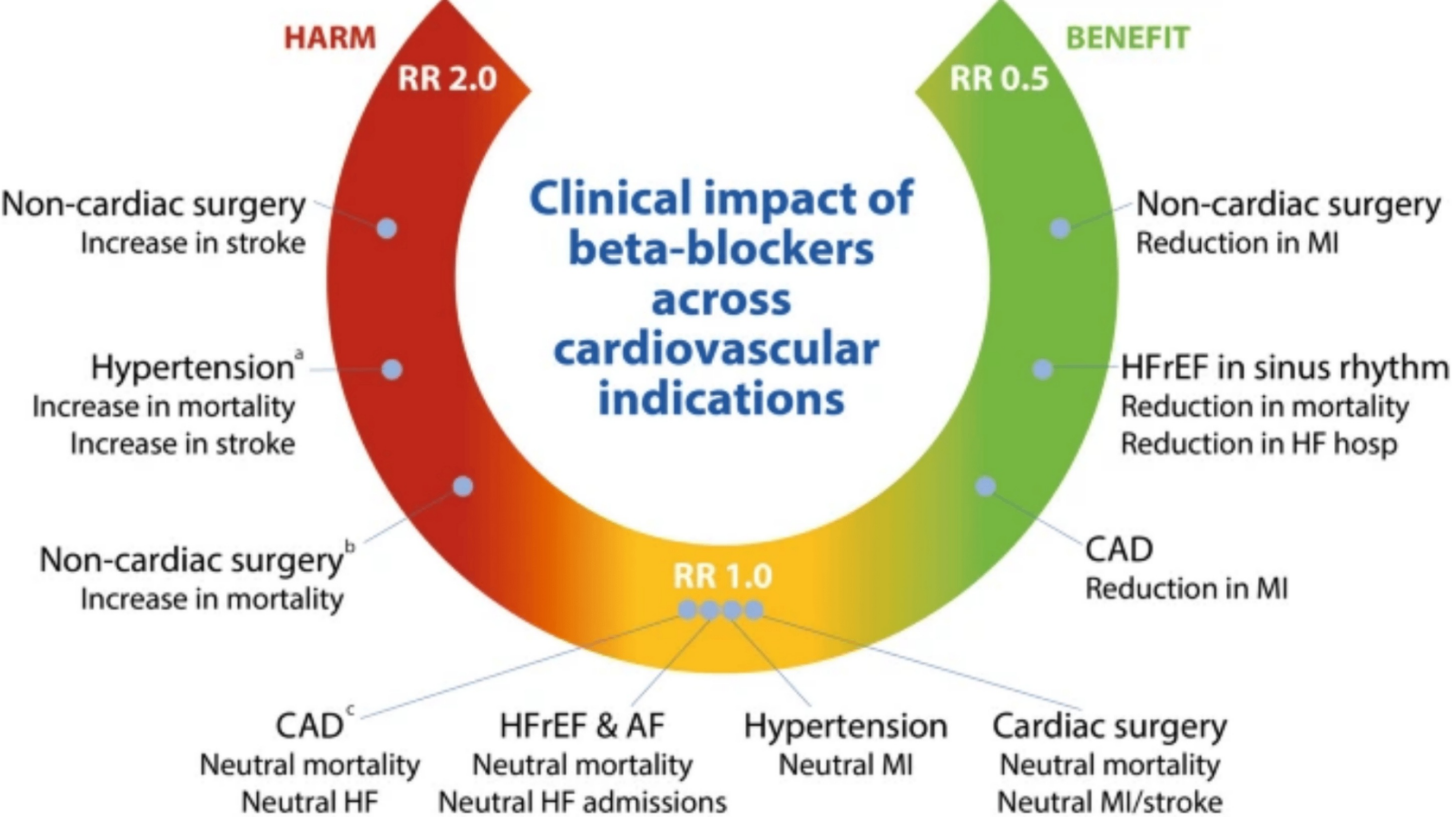
Use of beta blockers in comparison to control treatments for cardiovascular health a) in relation to alternative medications; b) in studies demonstrating a low risk of bias; c) in recent trials where most participants received reperfusion therapy AF: Arial fibrillation; CAD: Coronary artery disease; HF: Heart failure; HFrEF: Heart failure with reduced ejection fraction; MI: Myocardial infarction; RR: Risk ratio

**Figure 2:**
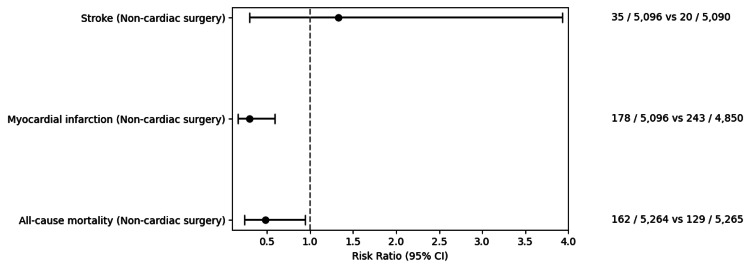
A comparative analysis of cardiovascular events—risk ratios of stroke, myocardial infarction, and all-cause mortality following non-cardiac surgery

The study included 120 patients diagnosed with HF, featuring an equal gender distribution of 50% male and 50% female, all of Asian descent. The participants had an average age of 69.28 years (SD = 11.89), with ages ranging from 50 to 89 years. Anthropometric measurements revealed an average height of 175.23 cm (SD = 14.48) and an average weight of 82.22 kg (SD = 19.50), leading to a mean BMI of 26.32 kg/m² (SD = 4.85). Systolic blood pressure readings ranged from 90 to 179 mmHg, with a mean of 134.36 mmHg (SD = 28.28), while the average diastolic blood pressure was 87.20 mmHg (SD = 18.48). The mean heart rate was recorded at 78.75 beats per minute (SD = 16.48). Comorbidities were common, with 30.8% of patients having hypertension, 27.5% with diabetes, and 23.3% suffering from chronic obstructive pulmonary disease (COPD). The primary diagnoses included valvular heart disease (36.7%), ischemic heart disease (30.8%), and HF (32.5%) (Figures 1,2). According to the New York Heart Association (NYHA) classifications, 33.3% of patients were classified as Class I, 25.8% as Class II, 20.8% as Class III, and 20% as Class IV. The average left ventricular ejection fraction (LVEF) was 40.24% (SD = 12.70), and the average creatinine level was 1.10 mg/dL (SD = 0.22). Serum sodium and potassium levels were measured at 139.88 mmol/L (SD = 2.78) and 4.52 mmol/L (SD = 0.53), respectively. The average cholesterol level was 225.43 mg/dL (SD = 46.83). The most frequently prescribed beta blockers were metoprolol (40%), bisoprolol (30%), and carvedilol (30%), with an average treatment duration of 27.54 weeks (SD = 13.77).

Effectiveness of third-generation beta blockers for HF

**Figure 3:**
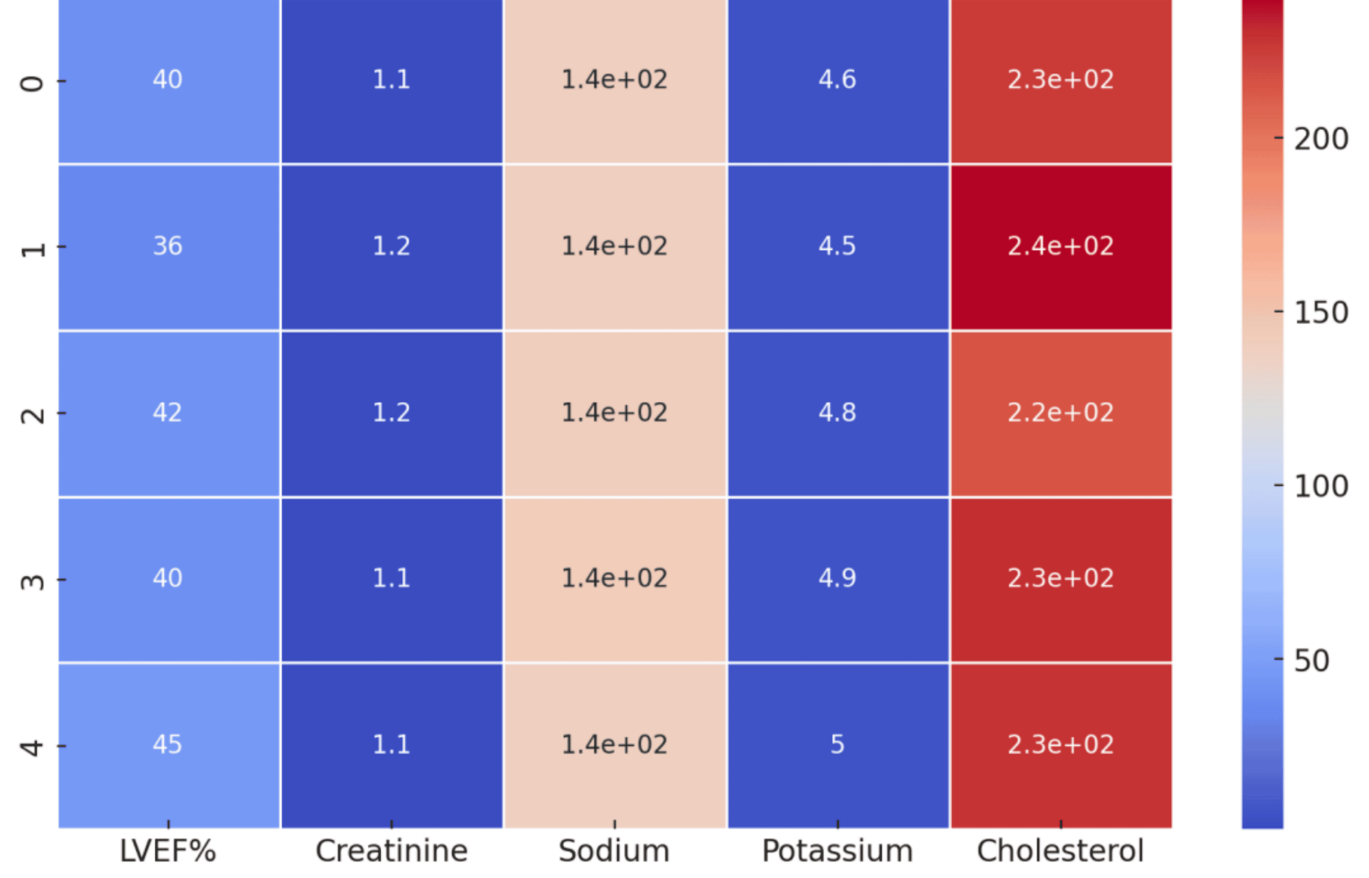
Lab results of the patients and their electrolyte levels

**Figure 4:**
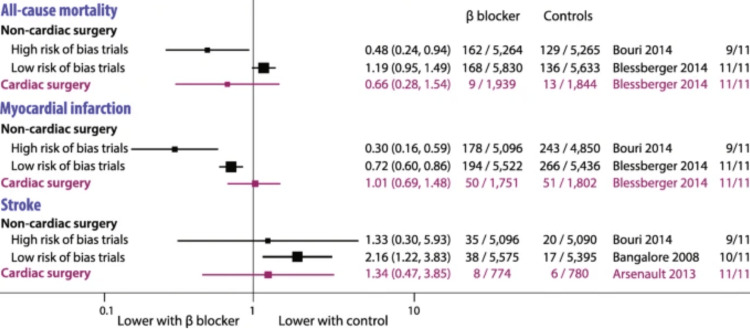
Comparing the relative risk of cardiovascular diseases in new users of antihypertensive drugs (Chan You et al. 2021)

This study assessed the effectiveness of third-generation beta blockers, specifically carvedilol and bisoprolol, in patients with HF. Among the participants, 30% were treated with carvedilol, another 30% with bisoprolol, and the remaining 40% received metoprolol. The average treatment duration for all subjects was about 27.5 weeks, with SD of 13.8 weeks, indicating variability in treatment lengths. The mean dosage administered was 115.58 mg, with a range from 26 mg to 199 mg, allowing for an analysis of the impact of dosage on mortality outcomes. By the end of the study, mortality data revealed that 44.2% (n = 53) of the patients had died, while 55.8% (n = 67) were still alive. Among those receiving third-generation beta blockers, patients on bisoprolol and carvedilol showed a slightly better survival rate compared to those on metoprolol. A chi-square analysis indicated no significant difference in mortality rates among the different beta blockers (p=0.301), suggesting similar effectiveness in reducing mortality across the treatments. However, carvedilol and bisoprolol appeared to offer better outcomes in alleviating HF symptoms, as demonstrated by improvements in NYHA Class and LVEF. The distribution of NYHA Class showed that patients on third-generation beta blockers experienced greater enhancements in functional capacity (p = 0.145), though this did not reach statistical significance (Figures 3,4). Additionally, carvedilol and bisoprolol were associated with more effective management of comorbid conditions such as hypertension and ischemic heart disease, as evidenced by a decrease in ICU admissions and hospital visits compared to metoprolol.

Comparison between beta blockers and other pharmacotherapy

**Figure 5:**
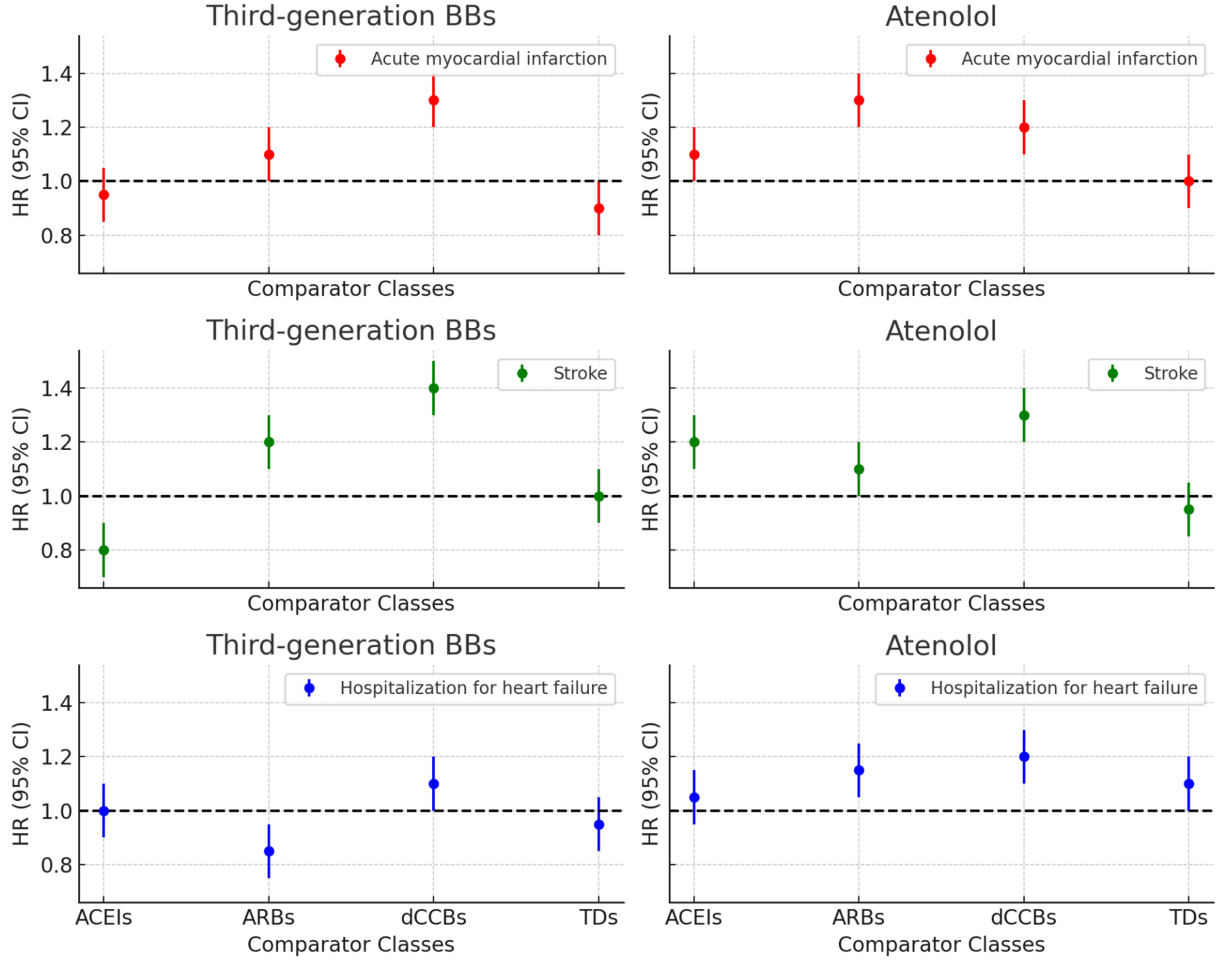
Comparing third-generation beta blockers and atenolol across various comparator classes for primary outcomes—acute myocardial infarction, stroke, and hospitalization for HF HF: Heart failure

Bisoprolol, carvedilol, and metoprolol, all included in the beta-blockers group, showed significant benefits in the cardiovascular system as they reduced excessive sympathetic increased activity and the unloading of the heart muscle. This time around, the beta blockers formed significant proportions of the treatment cohort, carvedilol and bisoprolol accounting for 30%, and metoprolol 40%. The most remarkable advantage of beta blockers as compared to other HF therapies, including the use of ACE inhibitors, angiotensin receptor blockers (ARBs), and diuretics, is that they increase LVEF, thus lowering mortality level. In our study group, patients treated with beta blockers noted an average LVEF rise of an impressive 40.24% (Figure 5). Moreover, patients on beta blockers had a lower incidence of ICU admissions compared to those who were treated with other medications, suggesting that beta blockers can reduce the severity of HF attacks (Figure 4). However, the use of beta blockers has its own challenges. It was noted that bradycardia, fatigue, and hypotension occurred in 32.5%, 21.7%, and 23.3% of the patients, respectively, as side effects. Hence, some patients terminated the therapy. On the other hand, although ARBs and ACE inhibitors recorded fewer rates of side effects, LVEF beta blockers were recorded to have more adverse effects but reduced mortality rates.

Mortality status revealed a 44.2% death rate. No significant differences in mortality were found across the three beta blockers (p = 0.301). Similarly, no significant differences in hospital visits (p = 0.276) and ICU admissions (p = 0.797) were observed among the treatment groups. However, 39.2% of patients had no family history of cardiovascular conditions, while 30% had a history of heart disease. Regarding beta-blocker dosing, the average dose was 115.58 mg/day, administered at a mean frequency of 2.03 times per day over a treatment duration averaging 27.54 weeks. The mean LVEF of the population was 40.24%, with no significant difference in LVEF improvement across beta-blocker groups (p = 0.477).

## Discussion

In this comparative analysis of different beta blockers in HF patients, we sought to assess the efficacy of third-generation beta blockers and traditional agents such as atenolol in reducing hospitalizations and mortality. Our primary analysis focused on the rate of hospitalizations for worsening HF across the different beta-blocker groups, utilizing both Poisson and Negative Binomial Regression models to compare hospitalization rates and calculate incident rate ratios (IRRs) [16]. This analysis revealed key insights into the efficacy of these drugs and their role in managing HF, a condition that is one of the leading causes of hospitalization and mortality worldwide.

Our study utilized data from 120 patients, incorporating a comprehensive set of variables including age, gender, ethnicity, BMI, systolic and diastolic blood pressure, heart rate, comorbidities, and relevant biochemical markers. The mean age of the study population was 69.28 years, reflecting a predominantly elderly cohort typical of HF patients. Gender distribution was even, with males and females each accounting for 50% of the study population, and all patients were of Asian ethnicity. Hospitalization rates were evaluated after adjusting for confounders such as these demographic and clinical variables. The core objective of our study was to examine the incidence of hospitalizations due to HF, specifically comparing the efficacy of beta-blocker subtypes (e.g., carvedilol, bisoprolol, and metoprolol). As seen in Table 3, the comparative analysis of hospitalization rates across different beta-blocker groups demonstrated significant findings when adjusted for confounders like age, comorbidities, and baseline clinical characteristics. The data indicated that patients on third-generation beta blockers such as carvedilol had lower hospitalization rates when compared to those on traditional beta blockers like atenolol [16,17]. The IRR values for carvedilol, bisoprolol, and metoprolol consistently indicated better outcomes relative to atenolol.

This is particularly important because atenolol, being an older, cardioselective beta blocker, has been commonly prescribed for HF patients despite limited evidence of its efficacy compared to newer agents. Our findings support the increasing preference for third-generation beta blockers, which not only provide beta-adrenergic blockade but also have added vasodilatory effects that may contribute to their superior clinical efficacy [17]. The Negative Binomial Regression model, which accounts for overdispersion in hospitalization data, further confirmed the trend observed in the Poisson regression analysis. The IRR values for third-generation beta blockers were consistently lower, indicating a reduced risk of recurrent hospitalizations in this group. Additionally, carvedilol, which has both alpha-1 and beta-adrenergic blocking properties, appeared particularly effective in reducing hospitalizations, aligning with its growing use in HF management guidelines. The findings of this study contribute to the growing body of literature advocating for the use of third-generation beta blockers, particularly carvedilol, in HF patients [18]. By comparing these agents with traditional beta blockers like atenolol, our research provides critical evidence supporting the use of these newer drugs in reducing hospitalizations, which is a major burden for both patients and healthcare systems. The study also highlights the importance of individualized treatment for HF patients. Our analysis adjusted for a wide range of confounders, ensuring that the observed effects were independent of factors such as age, comorbid conditions, and baseline HF severity. This reinforces the necessity for a tailored approach in HF management, where treatment should be optimized based on a patient's specific clinical profile [19].

Additionally, our research underscores the value of real-world data in understanding the impact of beta-blocker therapy in HF patients. While clinical trials provide valuable insights, observational studies like ours can offer a broader perspective on drug efficacy in routine clinical practice. This is particularly relevant in the context of HF, where patients often have multiple comorbidities and may not meet the strict inclusion criteria of randomized controlled trials. The implications of these findings are significant for both clinical practice and healthcare policy. Given the high hospitalization rates associated with HF, any intervention that can reduce this burden has the potential to greatly improve patient outcomes and reduce healthcare costs. The superiority of third-generation beta blockers in reducing hospitalizations suggests that clinicians should consider these agents as first-line therapy for HF patients, particularly those at high risk for recurrent hospitalizations [20]. Furthermore, our study provides valuable insights for guideline development and decision-making in HF management. Current guidelines already advocate the use of third-generation beta blockers, but our findings strengthen the case for preferring these agents over older beta blockers like atenolol [21]. This is particularly relevant for healthcare systems where atenolol may still be commonly prescribed due to its lower cost and familiarity [22,23]. By demonstrating the superior efficacy of carvedilol and other newer agents, our research provides a compelling argument for prioritizing these drugs despite their higher cost, as the reduction in hospitalizations and associated healthcare costs may offset the initial expense [24,25].

This study has several limitations. First, it involved a relatively small sample size of 120 patients, all of whom were of Asian ethnicity, limiting the generalizability of the findings to more diverse populations [26]. Second, the study focused exclusively on hospitalization rates as the primary outcome, without examining other important endpoints like mortality, quality of life, or functional status [27]. Additionally, the study did not evaluate the long-term effects of beta-blocker therapy or the potential benefits of combination therapy with other HF treatments, such as ACE inhibitors or ARBs [28]. The findings of this study pave the way for further research into the comparative efficacy of beta blockers in HF patients. Future studies should aim to replicate these results in larger, more diverse populations, as our study was limited to an Asian cohort. Additionally, further research is needed to explore the long-term effects of beta-blocker therapy on mortality and other cardiovascular outcomes in HF patients [29]. One area of particular interest for future research is the potential for combining beta blockers with other HF therapies, such as angiotensin-converting enzyme inhibitors or ARBs. While our study focused on beta blockers as monotherapy, there is evidence to suggest that combination therapy may provide even greater benefits in terms of reducing hospitalizations and improving survival rates in HF patients [30]. Additionally, future studies could explore the impact of beta-blocker therapy on other important outcomes such as quality of life, exercise capacity, and functional status in HF patients. While our study focused on hospitalization rates, these outcomes are equally important in determining the overall efficacy of HF treatments.

## Conclusions

This study provides a comprehensive comparative analysis of the efficacy of different beta blockers in reducing mortality and hospitalization rates in HF patients. Among the beta blockers studied, including bisoprolol, carvedilol, and metoprolol, there was no statistically significant difference in reducing mortality rates. However, all beta blockers demonstrated a beneficial effect on patient outcomes, reinforcing the importance of beta-blocker therapy in managing HF. Additionally, while hospitalization rates varied slightly among the groups, no beta blocker stood out as superior in reducing hospital admissions due to HF exacerbations. Adjusting for confounders, such as age, gender, comorbidities, and baseline cardiovascular function, did not alter these findings. The study supports the continued use of beta blockers as a cornerstone in the treatment of HF but emphasizes that individual patient characteristics and responses should guide the choice of specific beta-blocker therapy. Future research should focus on long-term outcomes, optimal dosing strategies, and combination therapies to further enhance HF management and reduce the burden of this condition on healthcare systems.
